# A qualitative study of the work experiences of midwives performing obstetric ultrasound in Norway

**DOI:** 10.1186/s12884-020-03333-9

**Published:** 2020-10-21

**Authors:** Magnhild Reiso, Berit Langli, Eva Sommerseth, Aud Johannessen

**Affiliations:** 1 Department of Clinical and Molecular Medicine, Faculty of Medicine and Health Sciences, Norwegian University of Science and Technology (NTNU), P.O. Box 8905, NO-7491 Trondheim, Norway; 2 Faculty of Health and Social Sciences, University of South-Eastern Norway (USN), P.O. Box 235, NO-3603 Kongsberg, Norway

**Keywords:** Experience, Midwifery practice, Midwives, Norway, Pregnancy, Prenatal care, Qualitative research, Ultrasonography

## Abstract

**Background:**

Performing obstetric ultrasound is part of midwifery practice in Norway. Knowledge of these midwives’ working situation can enhance understanding of what their work involves and the challenges they encounter in their practice. The aim of this study was to gain insight into how midwife sonographers perceive their work in obstetric ultrasound.

**Methods:**

A qualitative study with individual interviews was conducted in 2018. Midwives (n = 13) with a postgraduate ultrasound qualification who performed obstetric ultrasound in private clinics and/or the public health sector were included. All four regional health authorities in Norway were represented. The data gathered were analysed using content analysis.

**Results:**

The analysis resulted in three main themes. (1) Working as a midwife sonographer involves a holistic approach. By practising their competence, in both midwifery and sonography, they could answer questions and reassure pregnant women. The participants also had a feeling of great responsibility in their work. (2) Being part of a professional environment in obstetric ultrasound was important for professional interaction, belonging and learning. (3) Developing and maintaining competence as a midwife sonographer had a positive influence on midwives’ motivation and confidence, and allowed for more variety in their work.

**Conclusions:**

Holistic care of the pregnant woman, her partner and the unborn baby was an important part of the participants’ work. They wanted to meet colleagues within their field, develop their expertise and have influence over their work situation. Organizational factors seemed to affect the participants’ overall ability to practise their skills and thus also their job satisfaction.

## Background

Ultrasound is considered to be a beneficial tool in prenatal care, playing a central role to reveal pregnancy-related complications and optimize pregnancy outcomes [1]. In Norway, all pregnant women are entitled to one ultrasound examination in gestational week 17–19 [2]. This examination is offered at any level of public maternity care. The purpose of the examination is to determine the expected date of birth, the number of fetuses, the location of the placenta, and to observe the fetal anatomy and development [3]. This examination is one of eight recommended consultations in the prenatal care programme [2]. Any additional examinations are offered by public health care if clinically indicated [3]. Ultrasound is also a tool to estimate fetal weight and growth, the amount of amniotic fluid and to confirm the fetal position. Ultrasound examinations that are not clinically justified are not eligible for reimbursement.

In Norway, the ultrasound examination in gestational week 17–19 is mainly performed by midwives with a postgraduate ultrasound qualification. In the following text, these midwives are referred to as “midwife sonographers”. A midwife sonographer in Norway has completed the Postgraduate Ultrasound Education for Midwives at the Norwegian University of Science and Technology (NTNU), which is a one-year full-time course. By 2019, 237 midwives had completed this course [4]. Graduated midwife sonographers are qualified to perform independent obstetric ultrasound examinations. They are especially trained to perform the examination in gestational week 17–19 and other clinically indicated examinations. Their work also involves providing physical and mental care to the pregnant woman and her partner [5]. They work in both public and private health care.

The Norwegian health service is organized into four regional health authorities [6], and public maternity care is divided into three levels: birth rooms, maternity wards and women’s clinics. This classification is based on quality requirements, such as available qualified personnel and preparedness. Each regional health authority has at least one centre for fetal medicine located at the women’s clinics. These specialized centres are part of the public health sector. Norway is a country with considerable geographical distances and many isolated areas. The health service has agreed to comply with recommendations for decentralized and differentiated maternity care [7]. Prenatal care is an important national preventative health programme [6], which aims to promote a healthy lifestyle and reduce maternal and infant morbidity and mortality. The programme can help to reveal pregnancy-related complications, where pregnant women with an increased risk of complications are identified and referred to a hospital or clinic with the necessary expertise. Based on a professional assessment, pregnant women will be referred to the level in maternity care that is best suited the needs of the mother and fetus.

A previous study by Edvardsson et al. [8] shows that midwives in Sweden acknowledged ultrasound as a vital tool in prenatal care, but felt that it could also lead to concern and ethical dilemmas. This is also emphasized by midwife sonographers in Norway in a recent study [1]. Although they experienced high demands on their operational and counselling skills, they described performing obstetric ultrasound as very satisfying work [1].

Midwife sonographers have a key role in referring pregnant women to a maternity facility with suitable expertise to address the needs of the pregnant woman and fetus. They often have large catchment areas, and work at a great distance from the nearest women’s clinics. At the time of this study, there were few previous studies to our knowledge that specifically described the work experiences of midwife sonographers in Norway. This study may contribute further insights into their work experiences. Knowledge of midwives’ work situation can enhance understanding of what their work involves and the challenges they encounter in their practice. This in turn can provide inspiration for organizational changes related to their work.

## Methods

### Aim

To gain insight into how midwife sonographers perceive their work in obstetric ultrasound in Norway.

### Study design

A qualitative study design was adopted to elicit knowledge of how midwife sonographers perceive their work. To this end, semi-structured individual interviews were conducted [9].

### Recruitment

Recruitment of participants took place in 2017 and 2018. To gain insight into how midwife sonographers perceive their work in obstetric ultrasound, we recruited midwife sonographers from different geographical areas, large and small hospitals, and private and public health care facilities. Seventeen different workplaces were contacted and purposively recruited to ensure variety in geographical location, and also in the annual number of births and level of maternity care. In order to recruit participants from public maternity clinics, the head of department or the immediate superior of the midwife sonographers was first contacted by telephone. These were then sent information about the study by e-mail and asked to pass this on to the midwife sonographers. Midwife sonographers in private clinics were contacted based on information accessed from the official websites of the clinics. A poster was created containing brief information on the study and contact details. The poster formed part of the recruitment of participants and was sent to the relevant heads of department and private clinics. The study is thus based on a purposeful sample in an attempt to achieve diversity and nuances in order to present a broad variety of participant experiences. The planning of the study, recruitment of the participants and the data collection were all performed in collaboration between the first and second authors (MR and BL) in connection with their master theses in midwifery.

### Participants

The inclusion criteria were the above-mentioned ultrasound qualification for midwives, current work in obstetric ultrasound in Norway and fluency in oral and written Norwegian. The sampling resulted in 16 midwife sonographers interested in participating. Three of these were excluded because of the desire to prioritize variety in size of maternity facility and geographical location.

### Data collection

Data collection took place during the first quarter of 2018. The first and second authors prepared a joint interview guide in order to answer their respective research questions. The interview guide contained four main questions (Table 1). All interviews started with the first question in the guide. Beyond that, the order of the other questions varied depending on what the participants said. The first two questions in the interview guide formed a basis for fulfilling the aim in this part of the study, whereas the last two questions were highlighted in the master’s thesis written by the second author. Follow-up questions were based on what the participants said during the interview. The interviews were distributed between the two authors MR and BL.

**Table 1:** Overview of the questions in the interview guide

• How do you find working as a midwife sonographer?
• What do you think of your work tasks as a midwife sonographer?
• How do you experience situations when you discover deviations during an ultrasound examination?
• How do you find communicating unexpected findings?

The appropriate number of participants for the study was subject to continuous evaluation. Further data collection after 13 interviews was considered unlikely to add new information of significance based on the aim of the study [10]. Nine interviews were performed face to face, while three were conducted by telephone. All interviews were recorded using a Dictaphone and stored as audio files. The interviews lasted between 40 and 77 minutes (mean 55 minutes). The audio recordings were transcribed verbatim within two weeks by the interviewer. A quality control check was subsequently performed by the interviewer/transcriber. Demographic data were obtained from the participants prior to the interviews.

### Data analysis

Systematic text condensation was the analysis method employed. This involves a thematic cross-case analysis of qualitative data and was performed in the four main steps (Table 2) described by Malterud [9]. In this process, these four steps as described in Table 2 (Themes and Sub-themes) were performed through discussions among all authors.

**Table 2:** The method of systematic text condensation as described in four steps

1. Read all the interviews to gain an overall impression. This overall impression is summarized in four to eight preliminary themes.
2. Identify meaning units in the data, separate relevant text from irrelevant, and sort the meaning units into code groups.
3. Sort the content of each code group into two or three subgroups. The meaning units in each subgroup are abstracted into condensates in the first person.
4. Each condensate in each code group is summarized as an analytical text and validated by re-reading the original transcripts.

## Results

The participants represented 13 different workplaces and were all female. All four regional health authorities in Norway were represented. Five participants worked in private clinics, either full-time or in combination with a position in the public health sector. The remaining eight worked exclusively in public health care. The number of births in the various facilities ranged from fewer than 300 to more than 4000 annually. The participants’ ages varied from below 40 to above 60 years (mean 48 years). Their experience of working as a midwife sonographer varied from fewer than five to more than 20 years (mean 10 years). All of the midwives working in private clinics had work experience as midwife sonographers from the public health sector.

The analysis resulted in three main themes and seven sub-themes, all of which described how the participants perceived their work in obstetric ultrasound, as shown in Table 3.

**Table 3:** Overview of the main themes and sub-themes

Main themes	Sub-themes
**I:** Working as a midwife sonographer involves a holistic approach	A good examination is holistic, not merely technical
	Being a midwife sonographer involves a strong feeling of responsibility
**II:** Being part of a professional environment in obstetric ultrasound	Collegial cooperation: reassuring to have a second opinion
	Being a midwife sonographer can be a bit lonely
**III:** Developing and maintaining competence as a midwife sonographer	Gaining knowledge creates motivation and confidence
	Variation in tasks is important to maintain competence and provide variety
	Influence on one’s own work situation

### Working as a midwife sonographer involves a holistic approach

#### A good examination is holistic, not merely technical

It was important for the participants to provide holistic care for the pregnant woman, her partner and the unborn baby, and thus not to be merely concerned with the technical aspects of the ultrasound examination. This was expressed independently of whether they worked in private clinics or the public health sector. They emphasized their desire to use their knowledge during the examination, and the importance of this. Their competence in both midwifery and sonography enabled them to calm and reassure pregnant women and they thus also felt that their care was holistic. The participants found that the examination in gestational week 18 was often the first encounter with a midwife for the pregnant woman and her partner and the couple had many questions. The participants were not only able to answer questions regarding the ultrasound examination itself, but also questions related to pregnancy, fetal development, the birth and postnatal period. It was important for them to reassure the couple by involving them and explaining what was being done during the examination. They wanted to give the couple a good start as parents and a pleasant experience. Working as a midwife sonographer was described as positive, satisfying and rewarding. It was interesting to perform ultrasound and medical imaging, but communication with couples was also very inspiring. The actual ultrasound examination took place in the same way each time, but the interaction with the couples was always different. The participants emphasized the value of using their clinical experience and having a holistic approach when meeting expectant mothers. Talking with pregnant women about their lifestyle and previous pregnancies and detecting factors of importance for further follow-up care were considered important aspects of their work.

In addition, the participants found that some pregnant women could be nervous about the ultrasound examination, while others seemed to have a somewhat unrealistic expectation about the content of the examination. In such cases, their task was to normalize the situation, inform the couple and create realistic expectations before starting the examination. They felt that providing information about all the possible abnormal findings that an ultrasound examination could reveal would make the couple feel insecure if this information was not relevant to their situation.

It was challenging to communicate deviations to expectant parents. In such situations, they found that the couple had many questions that they could not always answer directly. They tried to be honest without being too direct, but neither did they gloss over abnormalities. They gained confidence in such situations with experience. They talked to the couple about what they found difficult and supported them with their presence. The importance of empathy and caring in their work was highlighted, irrespective of whether they worked in private or public health care. Maintaining contact with the couple in cases of adverse findings was considered a natural part of care. Procedures for further contact varied among the participants. It was also rewarding to interact with couples in such situations, and one of them said:

*‘If you’re going to do a good medical examination, I think you have to see the whole picture, it’s not just a technical thing.’ (Interview 1)*.

#### Being a midwife sonographer involves a strong feeling of responsibility

Being a midwife sonographer was described as a great responsibility. Part of their job was to detect deviations that could have important consequences and they reported being “afraid of overlooking” such deviations. They experienced high expectations and increasing demands on their ability to discover abnormal findings, in particular when examining the fetal anatomy in gestational week 18. The feeling of responsibility was especially emphasized in relation to the examination of the fetal heart, in addition to fetal growth and post-term pregnancies, since their assessment could determine where the woman would give birth. Participants who worked in smaller maternity facilities emphasized their responsibility in referring pregnant women to the correct level of maternity care to give birth. They were terrified at the thought of overlooking something that meant that the woman should have been referred to a more advanced level.

The participants had high expectations of themselves, but also described the expectations as unrealistic. They said that everyone would eventually experience overlooking something; it was described as part of the work of a midwife sonographer. Nevertheless, to have overlooked deviations and thus given couples stress in such situations was described as challenging, involving a self-interrogation where they asked themselves why they had not seen the deviation and looked for possible reasons.

The participants also emphasized that they were responsible for normal pregnancies and were not meant to diagnose. If something was abnormal or they were unsure about their findings, they would readily refer the woman to experts at a higher level for a new assessment. This was described as their duty and decisive for the care of the woman and fetus. The participants had to decide whether findings were normal or not and found uncertain findings to be challenging. The situation itself was described as “here-and-now”, where they had to make a quick decision. Referring a pregnant woman for a new assessment, only to be told that everything was normal, was perceived as one of the most difficult situations. In such cases, they felt they had made the woman unnecessarily anxious. Findings of soft markers were mentioned as an example, as one participant described:

*‘Let’s say you have a soft marker. You have things like that where you think, “Shall I just ignore it, or should I refer?” because that’s my decision. But then you see things that make you think, “Well, this is a good sign”. Having everyday decisions like that, that’s hard and maybe that’s part of what I take home with me. It’s all up to me, and I notice that I can’t shake it off, that responsibility. Because I know that as soon as I say something, I’ve messed up that pregnancy.’ (Interview 13)*.

### Being part of a professional environment in obstetric ultrasound

#### Collegial cooperation: reassuring to have a second opinion

The opportunity to consult a colleague during the ultrasound examination was appreciated. The participants found it reassuring to have a colleague who could perform the examination with them if needed and confirm their assessment. However, some participants working in smaller facilities or private clinics had no colleagues at their workplace to consult. In such situations, they were able to contact experts at the centres for fetal medicine to discuss their findings, a reassurance they described as “worth its weight in gold”. Respect, trust, understanding and helpfulness were words they used to describe this cooperation. One midwife sonographer said:

*‘It’s the responsibility of being alone in such a small place, I’m the only one looking ... I miss a colleague, so I could say “Could you take a look with me, let’s discuss this together”.’ (Interview 5)*.

#### Being a midwife sonographer can be a bit lonely

Being a midwife sonographer was described by some participants as lonely and as quite a distinctive field of work. Some participants working in the public sector had superiors who had limited knowledge of obstetric ultrasound and understood little of what their work implied. They would have benefitted from greater opportunity to share their experiences with other midwife sonographers, discuss their field of work and hear how others performed their work. Being in a group of colleagues was a rewarding learning experience. Several participants missed professional discussions and one said:

*‘That’s kind of the way it is, being a midwife sonographer, you feel a bit lonely. Being a midwife sonographer, that’s a kind of competence and professional responsibility that you can’t just discuss with any other midwife.’ (Interview 9)*.

### Developing and maintaining competence as a midwife sonographer

#### Gaining knowledge creates motivation and confidence

Knowledge acquisition was considered necessary to create motivation and confidence in the participants’ work. They expressed a prominent need and desire to gain further knowledge of the field. However, the opportunities to address this varied among the participants working in the public sector. It was a challenge to keep updated in their profession, which was especially emphasized by participants who worked alone as midwife sonographers. The symposiums held by the Norwegian Society for Diagnostic Ultrasound in Medicine were mentioned as useful opportunities to keep updated. Discussions about specific cases were highlighted as particularly useful. Midwife sonographers working in smaller facilities expressed a desire for mandatory study visits to centres for fetal medicine. They felt that these visits enriched their professional knowledge and improved the quality of their work. The desire for further education in obstetric ultrasound was also highlighted.

Independently of where they worked, the participants wished for feedback on their work, both positive and negative. They felt that they could all learn from feedback on cases where individual midwives had overlooked something abnormal and some called for a better internal system for such feedback. In addition, they found it of great value to receive discharge summaries after referring pregnant women with questions about abnormal findings. To see the conclusions was very informative and increased their confidence in their work. Participants who did not receive discharge summaries experienced this as a “missing link”.

#### Variation in tasks is important to maintain competence and provide variety

The participants also stated that different types of obstetric ultrasound examinations were important to maintain their competencies and provide variation in their work. Performing ultrasound examinations in the first, second and third trimesters created work variety and was also necessary to determine normal development at the various stages of pregnancy. Participants who had been given considerable responsibility stated that this improved their confidence and that they enjoyed working autonomously. Others reported not having been given the opportunity to put into practice everything they had learned during their ultrasound education and had thus lost certain skills within their profession. Some of the participants working in large public hospitals mainly performed ultrasound examinations in gestational week 18, and described this as unsatisfactory and monotonous. Working in private clinics was mentioned as an alternative to provide variety, with a greater likelihood of performing ultrasound examinations in all trimesters of pregnancy. Education, knowledge and experience were all factors that enhanced their confidence, as one participant said:

*‘I see such a lot of them that I can tell if there’s anything abnormal ... you have an eye for it, because you see so many. That’s how it is with ultrasound, you know, you react when something deviates, because you’re used to seeing what’s normal. It’s all a matter of experience and large numbers.’ (Interview 12)*

### Influence on one’s own work situation

The participants also wanted to influence their work situation. Some participants working in public health care expressed a desire for more control over their working day and found they had little influence in decision-making. Having adequate time for each ultrasound examination was emphasized as an important priority among participants who had started their own private practice. When they had more time available, they reported being more satisfied, as they were able to fulfil their wish to work holistically. The ultrasound examination itself could be performed in half an hour, but the participants did not consider the examination complete until they had talked to the couple and answered any questions they might have. Several participants working in the public sector expressed a wish for more time for the consultation, and one of them had the following experience:

*‘Seen from the outside, it appears to be a very practical job, precisely because of the time. That means you don’t always feel you’re doing enough and that you’re compromising yourself and your ... identity as a midwife. There’s not much time for care ... and you may want to give a bit more care, in this job too.’ (Interview 13)*.

## Discussion

This section discusses the results of this study in the order in which they are presented in the results section.

Our study emphasizes that the work of midwife sonographers entailed a holistic approach to the fetus, the pregnant woman and her partner. The examination in gestational week 18 is stated by pregnant women in Norway to be the most important consultation during pregnancy [11]. In the present study, the participants described their work as rewarding and satisfying. They pointed out that their expertise enabled them to calm and reassure pregnant women and answer their questions, which may be expected to enhance the quality of this type of pregnancy consultation. Good quality in prenatal care means that the care addresses individual needs [12]. Such individualized care, accompanied by the opportunity to ask questions, an accommodating attitude, explanations and information, as well as sufficient time for the consultation, will impact the pregnant woman’s experience of the examination, as shown in several studies [13–15]. In a study by Edvardsson [16], midwives expressed concern that ultrasound may medicalize the pregnancy. Internationally, other professions often perform ultrasound examinations during pregnancy. It has been argued that midwives are well suited to perform the ultrasound examinations in gestational week 17–19, precisely because of their competence, communication skills and focus on psychosocial health and care [17].

The results of this study also show that the participants had the perception that pregnant women did not always have realistic expectations of the content of the ultrasound examination. Other studies have emphasized this [8, 16, 18, 19]. Further, our study shows that the participants felt that informing couples about all possible abnormalities that could be detected by ultrasonography might create unnecessary anxiety. For the same reason, pregnant women themselves have also stated in other studies that they do not want such information [11, 20, 21]. Expectant mothers appreciate pregnancy being considered a normal event [20], and preparation for an ultrasound examination in the form of information about possible abnormalities may adversely affect this. Furthermore, pregnant women have reported being aware that the examination cannot guarantee that their baby will be healthy, as shown in another study [11]. It would also seem unrealistic to expect that a couple’s reaction to adverse findings would be eliminated if they received advance information about the possibility of such findings. How well prepared pregnant women should be for an ultrasound is subject to debate. It should be possible to ensure both the joy of future parenthood and the medical objectives of the examination.

At an ultrasound examination, the midwife sonographer is the first person to experience the spontaneous reaction of the couple when abnormal findings are discovered. Our study shows that such situations could be challenging, as do other studies [1, 18, 22]. As previously described in the literature [18, 23], such situations leave little time to prepare how to communicate the bad news. Although pregnant women express a desire for quick answers in such situations, they also emphasize the importance of the quality of the information provided [24, 25]. Any unusual findings detected at the ultrasound examination often require further examination at a centre for fetal medicine. Although the participants found it challenging not to be able to give the couple immediate answers, it may also seem unrealistic to expect such a practice. The participants also highlighted that they appreciated supporting the couples in such situations, as described in a previous study [19]. Providing follow-up support to the couple was underlined by the participants as an important part of their work. Other studies show that such factors will have a positive influence on the couple’s experience [24, 25]. Following up the couple in such situations provides continuity in health care, which pregnant women also have called for in several studies [25, 26]. Based on the above, follow-up care of the couple should be considered an important aspect of the work of midwife sonographers. Our results show variation in the participants’ practice regarding follow-up care, which suggests that implementation of such a practice may be an important focus area for the future.

This study demonstrates that the participants experienced great responsibility and increasing demands as to what they should detect during ultrasound examinations. The fear of failing to detect deviations, or other factors of importance, during the ultrasound examination has also been described by midwives performing obstetric ultrasound in Norway in a recent study [1]. Many Norwegian maternity facilities are located at great distances from each other and have different requirements for expertise and preparedness. In this context, our study emphasizes the responsibility of midwife sonographers for referring pregnant women to the most suitable level in maternity care to give birth, in order to ensure optimal health care for mother and baby during childbirth and in the postnatal period. This responsibility was especially emphasized by participants who performed obstetric ultrasound in smaller facilities. Prenatal discovery of certain congenital heart defects is crucial for transfer of the unborn child to a higher level of care to ensure the necessary expertise and preparedness at birth, as several studies illuminate [27, 28], rather than an emergency transportation of a sick neonate [29]. This example clearly demonstrates the role of midwife sonographers in detecting fetal anomalies prenatally and underlines the importance of performing high-quality ultrasound examinations in all geographical areas and at any level of maternity care.

Findings of unclear significance were pointed out as challenging in our study and the participants emphasized their experience of worrying the couple unnecessarily in such situations. This has also been found in other studies [8, 16, 19, 30]. Findings of soft markers were mentioned as an example of such situations. Such findings may create unnecessary worry among pregnant women and affect their attachment to the unborn child [21, 31, 32]. In the study by Åhman et al. [21], women diagnosed with soft markers in pregnancy stated that they would have preferred not to have known, or were hesitant about receiving, this information. The clinical value and management of soft markers are described in a number of articles [33–35]. These vary as to the type of follow-up care recommended, and how far soft markers should be considered significant or normal variants. Information regarding the assessment and importance of observed soft markers has been shown to vary among Swedish clinics [36]. Assessment of clinically uncertain ultrasound findings in a “grey area” may be a stressful task, especially without clear guidelines on how to deal with such findings [8]. National Norwegian guidelines could enhance the confidence of midwife sonographers when assessing such situations and prevent pregnant women from worrying unnecessarily. This would also promote equal treatment of pregnant women, in line with the strong emphasis on fairness and providing safe health care in the Norwegian welfare state.

Our study shows that the participants referred pregnant women to experts at a higher level in cases of abnormal findings. This suggests that midwife sonographers have a well-established and well-functioning system when deviations are suspected. Good cooperation with centres for fetal medicine was reassuring and promoted professional discussions, which was especially mentioned by participants working in small maternity facilities and in private clinics. Being part of a professional environment was stated to be important for one’s professional development. Colleagues and education generate knowledge, and providing good care is dependent on collaboration and the exchange of experiences among health care professionals [37]. Our participants working in small facilities also expressed the desire for mandatory study visits to a centre for fetal medicine. Such schemes can provide professional development and updating, as well as promoting collaboration in health care. Some participants in our study found it lonely being a midwife sonographer and study visits may also enhance the feeling of belonging.

A further point emphasized by the participants was the desire to enhance their knowledge in the field. Pregnant women should have equal access to high-quality health services ([38], § 1–1) and in order to achieve this, practitioners must also develop professionally [37]. The challenge of decentralized health services is to maintain quality and strength in small professional communities. Financing of further education and participation in professional events will enhance the knowledge, skills and confidence of midwife sonographers, which again will improve the quality of health services.

To share experiences and learn from good results and adverse incidents are fundamental factors in enhancing quality and patient safety in health care [39]. The participants in our study wanted to receive discharge summaries and feedback related to their work, which may be expected to improve their work quality. The Health Personnel Act ([40], § 21a) stipulates that it is forbidden to acquire information unless this is justified in terms of health care for the patient. The Health Personnel Act [40, § 29c] permits information to be disclosed to health professionals referring the patient, if the purpose is quality assurance of health care or learning for health professionals. The results of this study suggest that this legislation is interpreted and handled differently in practice. Lack of feedback may be a practice that prevents workplace learning and constrains professional development and confidence, which may again adversely affect the quality of health care.

The study shows that the participants wanted variety in their work and the opportunity to use their holistic competence when meeting pregnant women for the ultrasound examination. Work variety and autonomy are factors that affect job satisfaction [41]. According to The International Confederation of Midwives [42], holistic care is an important part of midwives’ work. Time pressure can, however, limit the possibilities to provide holistic care as desired by pregnant women, thus adversely affecting the quality of health services. Our results show that the participants enjoyed their work as midwife sonographers, although their practice of the profession was subject to organizational factors that affected their job satisfaction. Further, our study shows that it was important for the participants’ motivation and well-being to be given work variety within obstetric ultrasound, to be able to influence their own work situation and to be allocated sufficient time for the ultrasound examination to provide holistic care. These factors were prominent, independently of where they worked. Despite the small number of participants in our study, there was a clear tendency that midwives who had chosen to work in private facilities had done so because they felt that this would give them greater opportunity to work holistically.

### Limitations of the study

Our professional background as midwife sonographers and expertise in the field may have enhanced our ability to ask the participants relevant follow-up questions in the interviews. At the same time, this may also have influenced the design of the study. The obstetric ultrasound environment in Norway is somewhat limited and we cannot ignore the fact that our familiarity with this environment may have influenced our data. In order to limit the use of participants with whom we had a close relationship, we carefully chose the workplaces to contact during recruitment, even though we were aiming for a purposeful sample. To accomplish our desire to include midwife sonographers living in outlying areas, three interviews were conducted by telephone, due to time and financial constraints, which again may have influenced the quality of the interviews. After completing the first telephone interview, we considered the data to be sufficiently rich and therefore conducted two further interviews by telephone. Further, to enhance trustworthiness, quotations have been presented in the text. In addition, data were analysed and discussed among the authors. Also, to provide greater transparency when translating and writing the research manuscript, the authors collaborated and a native English speaker made the necessary corrections [43].

## Conclusions

This study emphasizes that midwife sonographers found it important to provide holistic care to the pregnant woman, her partner and the unborn child, in addition to the technical aspects of the ultrasound examination. This affected the midwives’ job satisfaction. The participants enjoyed working autonomously. At the same time they found their work to involve great responsibility. Collegial cooperation, professional development and feedback were factors that influenced their motivation and confidence. They wanted work variety within obstetric ultrasound and allocation of sufficient time for examinations in order to maintain and practise their overall competence and reach their full potential. Based on our study, work variety and sufficient time for consultations are aspects that seem to require improvement in the public health sector. Organizational factors seemed to affect how the midwives could practise their profession and thus also their job satisfaction. The participants’ experiences and challenges in their practice did not seem to vary according to geographical location, but seemed to depend more upon whether they worked in private or public health care and in small or large facilities. Study visits for midwife sonographers to other hospitals, an internal feedback arrangement and allocation of sufficient time for ultrasound examinations could contribute to professional development and enhance both job satisfaction and the quality of health care provided. Further studies are needed to fully explore these factors.

## Data Availability

The datasets generated and/or analysed during the current study are not publicly available due to protection of individual participants’ privacy and confidentiality but are available from the corresponding author on reasonable request.

## Abbreviations

NTNU: Norwegian University of Science and Technology.
USN: University of South-Eastern Norway.
